# Alcohol's impact on fine motor skills: Insights from minimally invasive surgical simulation

**DOI:** 10.1016/j.heliyon.2024.e30099

**Published:** 2024-04-20

**Authors:** Daan J. Verhoeven, Bas H. Verhoeven, Sanne MBI. Botden, Ivo de Blaauw, Maja Joosten

**Affiliations:** a Radboudumc, department of surgery, Nijmegen, the Netherlands; b Radboudumc, Amalia Children's hospital, Nijmegen, the Netherlands

**Keywords:** Minimally invasive surgery, Alcohol, Guidelines, Healthcare, Performance

## Abstract

**Background:**

Alcohol misuse among medical professionals poses a significant concern, and there is a lack of clarity in (inter)national guidelines regarding alcohol use during work. Moreover, there exists an insufficient body of research on the specific impact of alcohol on fine motor skills within the medical sector, specifically surgery. This study aims to investigate the impact of alcohol on fine motor skills in a minimally invasive surgical setting.

**Methods:**

A cross-sectional study was conducted at Lowland Science on August 19th, 20th, and 21st, 2022, during the Lowlands music festival in Biddinghuizen, the Netherlands. Participants were divided into five groups based on measured alcohol consumption. Exclusion criteria included drug use, prior surgical experience, being underage, and previous participation. The main outcomes were the number of correctly transferred rings during the PEG transfer task and the number of errors. Blood alcohol concentration (BAC) was measured using a breathalyser.

**Results:**

A total of 1056 participants were included in the study. The results indicated an inverse relationship between BAC levels and surgical performance, with higher alcohol levels associated with a decrease in performance (p = 0.023). However, there was no significant difference in the number of errors among the five groups (p = 0.597). The group with the highest alcohol consumption (BAC >0.08 %) exhibited significantly worse performance compared to the group with a BAC of 0.0 % (p = 0.002).

**Conclusion:**

This study uncovers a negative impact of increased alcohol intake on fine motor skills in a minimally invasive surgery simulation exercise. While there was no effect on the occurrence of errors. Professional medical organizations should reconsider and explicate their position on alcohol use in (surgical) healthcare.

## Highlights

• As The Blood Alcohol Concentration (BAC) rises, fine motor skills in surgical simulation exhibits a steady deterioration.
• This inverse relationship was seen in a diverse non-surgical population, including individuals of all ages and genders.
• The need for a critical evaluation of (inter)national guidelines concerning alcohol consumption in the context of (surgical) healthcare.

## 1 Introduction

Evidence suggests a high prevalence of alcohol misuse among physicians, specifically surgeons [[1], [2], [3], [4]]. Furthermore, there are grounds to assume that global alcohol consumption among healthcare workers has increased since the onset of the COVID-19 pandemic [[5], [6], [7], [8], [9], [10]]. In the presence of such a risk, what do the professional regulations dictate? Several professional medical associations forbid alcohol or strongly advise against its use during working hours (or just before) [[11], [12], [13], [14]]. Some of these recommendations are not so strict and leave room for interpretation [[12], [13], [14]]. It seems that some international, maybe cultural, differences exist. In several guidelines the following is mentioned “must not be under the influence of …”, “surgeons should not practice or operate when their performance is impaired by alcohol …” or “not work when your health is adversely influenced by alcohol”. The question arises how to determine when this is the case. It is well-established that there is an underestimation of the overall amount of alcohol consumed [15,16]. Additionally, there is a tendency to underestimate one's own alcohol use, which can potentially lead to hazardous situations [17,18].

Considering the shared responsibilities between pilots and surgeons, understanding the regulations governing pilots is interesting. Both professions demand precision, accuracy, attention to detail, effective teamwork, and stress management. Pilots, subject to regular testing, have alcohol limits set at 0.02 % in Europe and the UK, and 0.04 % in the USA [19,20]. Despite limited alcohol impact research on surgical skills, parallels from aviation studies suggest it can degrade instrument handling systems, and lead to overall performance decline [21,22]. This indicates potential risks for surgical safety. Globally, alcohol limits for driving vary among countries [23]. Western European countries typically set the standard at 0.05 %, with exceptions like England, Wales, and Northern Ireland, allowing 0.08 % [24]. In the USA, the accepted limit is 0.08 % across all states [23,24]. Japan (0.03 %) and China (0.02 %) impose stricter limitations [23]. Despite the well-known dangers, a recent study in the Netherlands has unveiled that five percent of drivers participated in traffic over the last year with a higher level of alcohol than legally permissible [25]. This revelation not only raises concerns about road safety but also prompts a reflection on the parallels that may exist in the realm of medical practice. Much like the persistent occurrence of impaired driving despite its known risks, the potential for practicing medicine under the influence becomes a thought-provoking issue, particularly as guidelines in countries like the Netherlands and the UK hint at the possibility of such scenarios [11,13].

For the past three decades, minimally invasive surgery (MIS) has consistently stood as the gold standard for a wide array of surgical procedures, encompassing both elective and acute interventions [[26], [27], [28], [29]]. However, the impact of alcohol on the intricate motor skills required for these procedures has received limited attention in the research literature [30,31]. Despite the scarcity, existing guidelines exhibit a lack of clarity, universality, and room for interpretation. Understanding the influence of alcohol on fine motor skills is crucial, especially in minimally invasive surgery. Examining its impact on precision and dexterity in procedures provides insight into potential consequences on surgical performance.

## 2 Methods

### 2.1 Study design

We employed a cross-sectional design to investigate the impact of alcohol consumption on the performance of a minimally invasive surgical skill. The study was conducted at Lowlands Science, a dedicated area within the Lowlands music festival in Biddinghuizen, the Netherlands, held from August 19th to 21st 2022. The study was approved by the ethical commission of Nijmegen and Arnhem (casenumber 2020–6289).

### 2.2 Participants

**Fig. 1:**
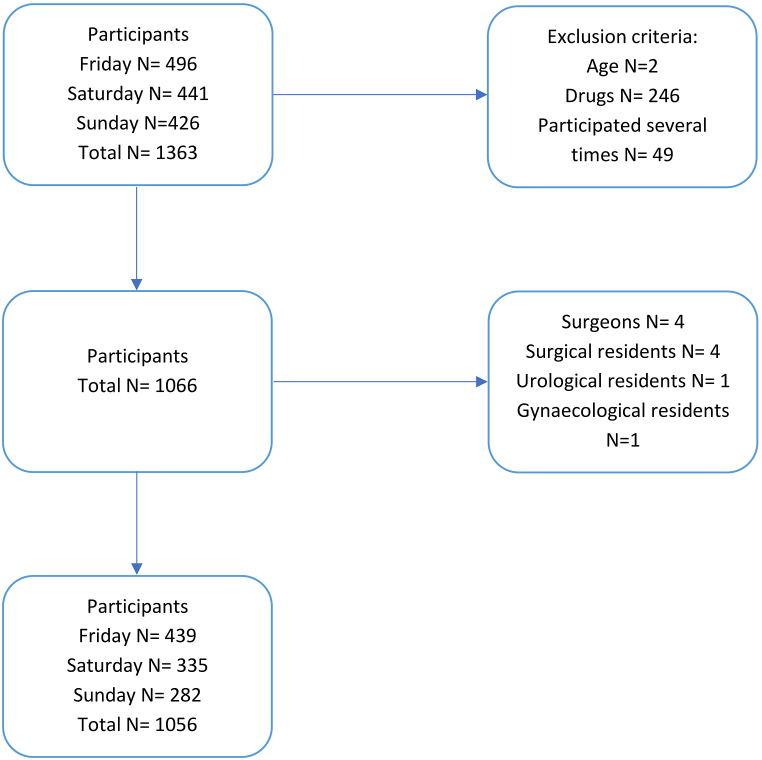
Flowchart of the exclusion process.

Study participants were recruited from festival attendees at Lowlands Science. All individuals provided voluntary and informed consent for the anonymous use of their data for scientific research purposes. Exclusion criteria consisted of drug use, previous participation, incapacity, or participants under the age of 18 (Fig. 1). Individuals with a background in surgery, such as surgeons or surgical residents, including urology, orthopaedics and gynaecology residents were excluded. Participants were allotted to five different groups based on measured blood alcohol concentration (BAC).

**Fig. 2:**
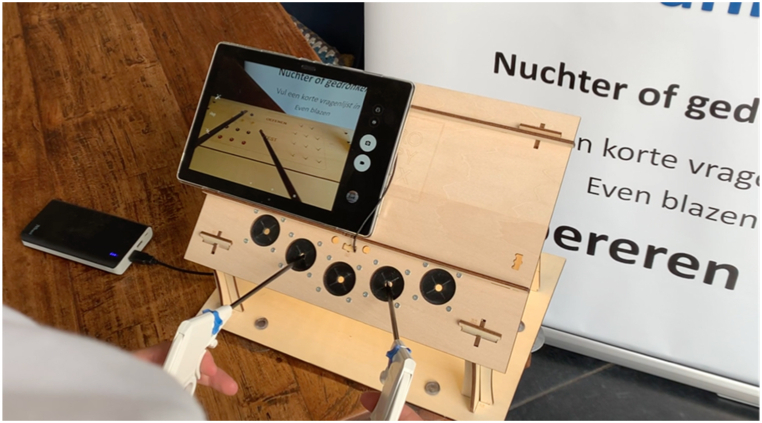
The LaparoscopyBoxx.

*Materials* Participants completed a minimally invasive surgical task on a simulator for minimal invasive surgery, the LaparoscopyBoxx (Outside the Boxx, Nijmegen, the Netherlands) [32]. This low-budget wooden box training was developed to be utilized at home, highlighting accessibility and effectiveness [33,34] (Fig. 2). It has a wooden front with three or five instrument ports and an opening in the centre of the top panel, which is designed for the camera of a tablet. A Lenovo P10 tablet with an Android operating system was used in this study. Different task boards could be fixed in the simulator by clicking them in the posterior pillars in the proper position. For this study, a task board specifically designed for a peg transfer was used [32,35]. Participants were provided with a 5 mm needle holder for their right hand and a grasper for their left hand. In this study, the Alcovisor Mercury breathalyser was used for alcohol concentration measurement. Each participant used a dedicated mouthpiece during testing. The Mercury is an European Commission and FDA-approved professional-grade alcohol meter [36]. The measurements were expressed in units or “part per mille” (grams of alcohol per litre of blood).

### 2.3 Task

**Fig. 3:**
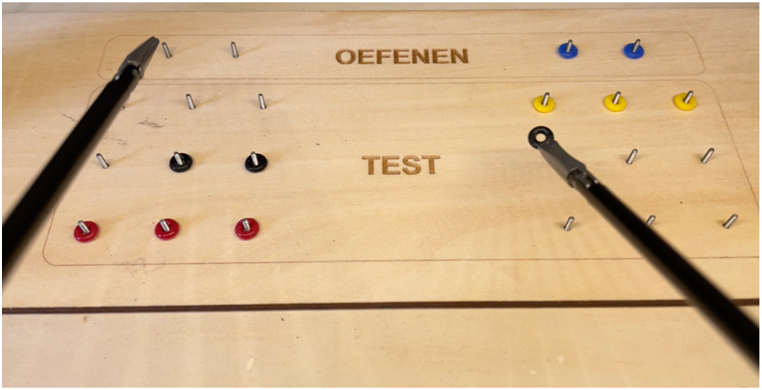
Laparoscopic task. Dutch translation: oefenen = practice.

Before the participants performed the basic peg transfer task, they viewed the instructional video and practiced for 30 s. The participants utilized this allocated time to familiarize themselves with the instruments. This included gaining a hands-on feel of how the instruments open and close, as well as understanding the movement of the instruments in the simulator through the fulcrum effect. During the familiarization, they were instructed to move two small silicone rings, initially positioned on pegs on the left side of the board, to two pegs on the right side and were allowed to use both instruments. Subsequent to the practice session, participants performed the task, involving the transfer of nine silicone rings from the left pegs to the right pegs. The task board, with a total of eleven rings (two from the practice session and nine from the task session) can be seen in Fig. 3. There was a 3 minute time limit. If all rings were moved to the right side within 3 minutes, participants transferred the rings back to the left side of the board. Therefore, the score could exceed nine.

### 2.4 Outcome measures

Performance was assessed by quantifying the accurate transfer of rings to the opposing side of the board, while dropped rings were deemed as errors. Alcohol concentration was measured in parts per thousand (g alcohol/L blood) using a breathalyser test. The recorded values were transformed into percentages, also known as the Blood Alcohol Concentration (BAC). This transformation was accomplished by multiplying the original measurement (units per thousand) by 0.1, resulting in the unit g alcohol/dL blood.

### 2.5 Protocol

The Lowlands science region was a designated area separate from the rest of the festival grounds, opened Friday through Sunday from 10 a.m. to 8 p.m. After obtaining informed consent, Researcher A facilitated the participants in completing the questionnaire covering sleep patterns, drug use and alcohol use. And conducted the Breath Alcohol Concentration (BAC) measurement, noting the recorded values on the front of the form. Subsequently, participants were guided to a separate area by Researcher B, where they viewed a brief instructional video on the peg transfer task and instrument use. Researcher B ensured that prior to engaging with the simulators, all participants comprehended the task, including the procedures for opening and closing the instruments. Following this, the practice session and the peg transfer test were administered. Meanwhile, Researcher C received the form from Researcher B, ensuring that only the back of the form, containing the (still empty) score assessment, was visible for researcher C. The results of the test were then recorded on the back of the form by researcher C, and securely stored in a locked box. This approach was implemented to prevent the assessors from having access to the variables on the front of the form. The importance of this procedure was communicated to the researchers beforehand.

### 2.6 Statistics

Statistical analysis was performed with IBM SPSS Statistics for Windows, version 27 (IBM Crop., Armonk, N.Y., USA). Legal limits with regard to driving under the influence of alcohol were used as cut-off values, because it is difficult to determine with substantiated arguments a limit value for operating on patients [23,24]. Participants were divided into five groups based on alcohol percentage: group 1 (0 %), group 2 (0.0–0.02 %), group 3 (0.02–0.05 %), group 4 (0.05–0.08 %) and group 5 (>0.08 %). An analysis of variance (ANOVA) was conducted to examine whether statistically significant differences exist among the various groups for continuous variables. A chi-square test was employed for ordinal variables. The Kruskal-Wallis test was chosen for variables that did not meet the assumptions for parametric testing when comparing groups. To investigate the presence of a progressive decline or trend across the groups, the Jonckheere-Terpstra test was employed [37]. Results with a p-value <0.05 considered a statistically significant difference. To account for multiple comparisons and control the overall type 1 error rate, the Bonferroni correction was applied to the pairwise comparisons [38].

## 3 Results

From the initial 1363 participants enrolled in the study, a total of 1056 individuals met the predefined inclusion criteria and were included in the final analysis (Fig. 1). Exclusion criteria were applied, resulting in the exclusion of 308 participants from the analysis. The primary reason for exclusion was drug-related factors (N = 246). The participant's ages exhibit a non-normal distribution, with a median age of 28 and an interquartile range (IQR) spanning from 25 to 32. There were more female participants than male participants (597 vs. 454), with five participants identifying themselves as non-binary. According to the chi-square test (Table 1), there is a significant difference in the proportions of men and women in the five different groups (p = 0.025). This disparity reflects well-established patterns in alcohol consumption, where men generally consume more alcohol than women [39]. Table 2 shows a significant difference in the distribution of fitness among the groups (p = 0.028). Nevertheless, gender does not impact the score (p = 0.897) or errors (p = 0.674). This holds true for fitness as well (p = 0.670, p = 0.913).

**Table 1:** Demographics.

	Total	Group 1 (0 %)	Group 2 (0.0–0.02 %)	Group 3 (0.02–0.05 %)	Group 4 (0.05–0.08 %)	Group 5 (>0.08 %)	P-value
Age (years)	28 (25-32)	29 (24-33)	28 (25-32)	28 (25-32)	28 (24-33)	28 (25-34)	0.948
*Gender*							0.025*
Male	454 (43.0 %)	164 (37,7 %)	86 (44.8 %)	122 (45.9 %)	42 (45.2 %)	39 (57.4 %)	
Female	597 (56.5 %)	271 (62.3 %)	105 (54.7 %)	143 (53.4 %)	49 (52.7 %)	29 (42.6 %)	
Other	5 (0.5 %)	0	1 (0.5 %)	2 (0.7 %)	2 (2.1 %)	0	
*Dexterity*							0.840*
Right	906 (85.8 %)	370 (85.0 %)	163 (84.9 %)	236 (88.1 %)	81 (87.1 %)	56 (82.4 %)	
Left	133 (12.6 %)	55 (12.6 %)	27 (14.1 %)	28 (10.4 %)	11 (11.8 %)	12 (17.6 %)	
Both	6 (0.6 %)	3 (0.7 %)	1 (0.5 %)	2 (0.7 %)	0	0	

Values stated in median (IQR) or numbers (column percentage). P-values were calculated using Kruskal-Wallis or Chi-square test*. A P-value <0.05 (displayed in italic) was considered significant.

**Table 2:** Sleep & fitness.

	Total	Group 1 (0 %)	Group 2 (0.0–0.02 %)	Group 3 (0.02–0.05 %)	Group 4 (0.05–0.08 %)	Group 5 (>0.08 %)	P-value
*Sleep quality*							0.138**
**Good**	186 (17.6 %)	76 (17.5 %)	29 (15.1 %)	46 (17.1 %)	18 (19.4 %)	17 (25.0 %)	
**Average**	604 (57.2 %)	258 (56.7 %)	109 (56.8 %)	159 (59.3 %)	52 (55.9 %)	26 (38.2 %)	
**Bad**	261 (24.7 %)	101 (23.2 %)	49 (25.5 %)	63 (23.5 %)	23 (24.7 %)	25 (36.8 %)	
*Fit*							0.028**
Tired	150 (14.2 %)	78 (17.9 %)	30 (15.6 %)	28 (10.4 %)	9 (9.7 %)	5 (7.4 %)	
Not fit nor tired	552 (52.3 %)	229 (52.6 %)	101 (52.6 %)	148 (55.2 %)	45 (48.4 %)	29 (42.6 %)	
Fit	331 (31.3 %)	124 (28.5 %)	58 (30.2 %)	86 (32.1 %)	35 (37.6 %)	28 (41.2 %)	
Hours slept	5.9 (1.6)	6.0 (1.5)	5.8 (1.5)	5.8 (1.6)	5.8 (1.6)	6.0 (1.9)	0.314
Times woken up	2 (1-3)	2 (1-3)	2 (0–3)	2 (1-3)	2 (0–3)	2 (1-3)	0.401*
*Game-experience*							0.266**
**None**	521 (49.3 %)	223 (51.3 %)	98 (51.0 %)	135 (50.4 %)	39 (41.9 %)	26 (38.2 %)	
**Reasonable**	363 (34.4 %)	148 (34.0 %)	64 (33.3 %)	90 (33.5 %)	37 (39.8 %)	24 (35.3 %)	
**Much**	160 (15.2 %)	59 (13.7 %)	27 (14.1 %)	40 (14.9 %)	17 (18.3 %)	17 (25.0 %)	

Values stated in mean (±), median (IQR) or numbers (column percentage). P-values were calculated using ANOVA, Kruskal-Wallis * or Chi-square test**. A P-value <0.05 (displayed in *italic*) was considered significant.

**Fig. 4:**
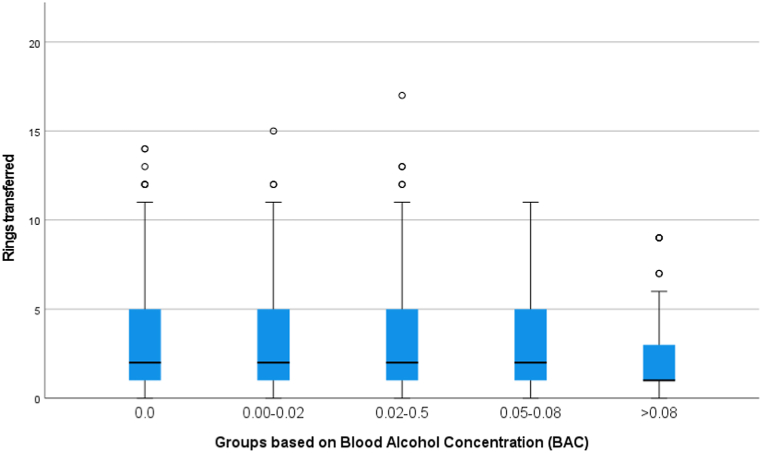
Boxplot of the performance in the different groups.

Of the participants, 476 (45 %) moved at least two rings, contrasting with 580 (55 %) who moved only one ring or none. Moreover, more than half of the participants 590 (56 %) dropped one or more rings during the exercise. A Jonckheere-Terpstra test was conducted to examine the presence of a direction or systematic change in performance scores across the ascending groups. The analysis included five groups, which were numbered based on increasing levels of BAC (Fig. 4). A statistically significant decline in surgical performance was detected as BAC levels increased among the ordered groups (p = 0.023). However, no significant association was found between the number of errors and alcohol consumption with the Jonckheere-Terpstra test (p = 0.597), suggesting that the number of errors did not significantly increase with higher levels of alcohol consumption (Table 3). To assess the performance differences among groups, we conducted Mann-Whitney U tests comparing the 0.08 % group with each of the other groups (0 %, 0.0–0.02 %, 0.02–0.05 and 0.05–0.08 %). The Bonferroni correction, used to control a Type I error rate of 0.05, yielded an adjusted significance level of 0.00625 (0.05/8). The comparison between the 0.0% and >0.8% groups revealed a significant difference in scores (p = 0.002), indicating that the 0.0% group performed better. However, no significant differences were observed in error rates between these two groups (p = 0.829). In the comparisons with the other groups (0.0–0.02 %, 0.02–0.05 % and 0.05–0.08 %), no significant differences were found regarding the scores (p = 0.061, p = 0.029, p = 0.016, respectively) and errors (p = 0.937, p = 0.597, p = 0.508, respectively).

**Table 3:** Scores.

	Total	Group 1 (0 %)	Group 2 (0.0–0.02 %)	Group 3 (0.02–0.05 %)	Group 4 (0.05–0.08 %)	Group 5 (>0.08 %)	P-value
Rings transferred	2.0 (1.0–5.0)	2.0 (1.0–5.0)	2.0 (1.0–5.0)	2.0 (1.0–4.5)	2.0 (1.0–5.0)	1.0 (1.0–3.0)	*0.023*
Rings dropped	1.0 (0.0–1.0)	1.0 (0.0–1.0)	1.0 (0.0–1.0)	1.0 (0.0–1.0)	1.0 (0.0–1.0)	1.0 (0.0–1.0)	0.597

Values stated in median (IQR). P-values were calculated using a Jonckheere-Terpstra test.

## 4 Discussion

The study revealed a clear link between alcohol consumption and fine motor skills in a minimal invasive surgical skill using simulation. As alcohol concentration increased, there was a noticeable declining trend in fine motor skill performance. These findings highlight the importance of considering alcohol intake carefully in surgical settings, as it can significantly affect the quality of technical procedures. It is possible that the decrease in performance had already started in the slightly more sober group (0.05–0.08 %) but was only evident in the group with a higher BAC (>0.08 %). Whether a threshold exists and where it would lie cannot be determined from our data. We anticipate that this varies heavily depending on the kind and difficulty of the task at hand. In addition, our research showed that higher amounts of alcohol do not necessarily lead to more errors made in this particular simulation exercise, not even at high alcohol levels (>0.08 %). Our results findings align with previous research by Dorafshar et al. which suggesting a performance decrease in basic laparoscopy tasks just above 0.08 % BAC in medical students [30]. Additionally, a study conducted by Kirby et al. suggests that consuming three or more glasses of wine is associated with decreased performance in four ENT surgeons [31]. However, they used glasses as a measurement rather than BAC making direct comparisons challenging [31].

As we reflect on these results, it becomes apparent that nations should thoroughly reassess and potentially revise their current guidelines and recommendations. In this revision process, introducing clear and objective rules, eliminating any ambiguity or room for interpretation, is crucial. Collaborating with experts in various fields, including psychology, physiology, and law, can contribute to a more complete formulation of guidelines. Moreover, exploring the feasibility of developing a universal international guideline addressing the complexities of alcohol consumption and its impact on fine motor performance is valuable. Public awareness campaigns and educational initiatives could play a pivotal role in promoting adherence to revised guidelines and fostering a broader understanding of the potential risks associated with impaired performance due to alcohol consumption.

In addition to technical proficiency, non-technical skills such as communication and teamwork play a crucial role in achieving excellence in surgical practice. These skills not only contribute to patient safety but also foster a sense of reassurance and well-being among patients [40,41]. Previous research indicates that increased alcohol consumption can have a negative impact on communication skills [31]. It may manifest in the form of inappropriate language use, difficulties recalling the names of surgical instruments, and delayed responses to communication cues [31]. This could potentially lead to hazardous situations.

Apart from the effects on performance, it should be mentioned that alcohol itself also has its share of downsides. It serves as a significant risk factor for the global burden of disease, contributing to various types of cancer, causes many deaths, injuries, and a whopping 11 % of healthcare costs [39,[42], [43], [44]]. Not drinking alcohol is the only amount that does not have a negative effect on your health [45,46]. The more alcohol consumed, the higher risk of disease or death [43]. Which does not even address the negative effects of alcohol on social life and psychological health [[47], [48], [49], [50]].

### 4.1 Strengths and weaknesses

A major strength of our study is the inclusion of a large and diverse group of participants. Previous research has only been done in small sample sizes and exclusively with healthy male participants [30,31]. However, in the present day, surgeons are not exclusively male nor completely healthy. The participants of this study were not surgeons and therefore not used to these kinds of exercises. The question is whether the effect of alcohol on minimally invasive procedures is the same for surgeons. In the ideal situation, a large group of willing surgeons to participate in the study is required. Obtaining approval from the medical ethics committee is expected to present serious and understandable challenges. Therefore, we consider our current festival population as the best alternative to study. Furthermore, it is crucial to acknowledge that the complexity of most surgical procedures extends beyond the specific simple task in this study. Factors such as teamwork dynamics, anatomical knowledge, procedural expertise, and stress management play pivotal roles in real surgical scenarios. Therefore, the simulation used in this study cannot be directly equated with the intricacies involved in medical practice operations. Another thing to keep in mind is that alcohol consumption was objectively measured, while drug use was assessed through self-reporting. Furthermore, there may be additional (personal) variables that could influence the successful execution of the task, variables which were not measured in our study. These might include factors like concentration (arc), exposure to distracting stimuli during the festival, dealing with frustration-tolerance and personal talent. This, however, remains unclear and research is needed to gain a deeper understanding of the intricate interrelationship of personal and environmental factors that contribute to good fine motor skills.

## 5 Conclusion

As alcohol consumption increased among participants, there was a corresponding decline in performance. Notably, performance deteriorated significantly beyond the 0.08 % BAC threshold when compared to the sober state. It is imperative for (inter)national guidelines in (surgical) healthcare to re-evaluate their stance on alcohol, striving for consistency and placing a premium on objectivity in their recommendations.

## Ethics statement

The study was approved by the ethical commission of Nijmegen and Arnhem (casenumber 2020–6289). All participants provided written informed consent for the use of their data for scientific research purposes.

Financial support and sponsorship: our study did not receive specific funding or sponsorship.

## Availability of data and materials

Data associated with the study has not been deposited into a publicly available repository. Data are available from the corresponding author on reasonable request.

## CRediT authorship contribution statement

**Daan J. Verhoeven:** Writing – original draft, Investigation, Formal analysis. **Bas H. Verhoeven:** Writing – review & editing, Supervision, Investigation, Formal analysis. **Sanne MBI. Botden:** Writing – review & editing, Supervision, Methodology, Conceptualization. **Ivo de Blaauw:** Writing – review & editing, Supervision, Methodology, Conceptualization. **Maja Joosten:** Writing – review & editing, Methodology, Investigation, Conceptualization.

## Declaration of competing interest

The authors declare that they have no known competing financial interests or personal relationships that could have appeared to influence the work reported in this paper.

## Abbreviations

MIS: Minimally Invasive Surgery
BAC: Blood Alcohol Concentration
IQR: Inter Quartile Range

## References

[1] Oreskovich M.R., Kaups K.L., Balch C.M., Hanks J.B., Satele D., Sloan J. (2012). Prevalence of alcohol use disorders among American surgeons. Arch. Surg..

[2] Bhattacharya K., Bhattacharya N. (2023). Alcoholism among surgeons-is it a hidden hazard?. Indian J. Psychol. Med..

[3] Medisauskaite A., Kamau C. (2019). Does occupational distress raise the risk of alcohol use, binge-eating, ill health and sleep problems among medical doctors? A UK cross-sectional study. BMJ Open.

[4] Wilson J., Tanuseputro P., Myran D.T., Dhaliwal S., Hussain J., Tang P. (2022). Characterization of problematic alcohol use among physicians: a systematic review. JAMA Netw. Open.

[5] Ramalho R. (2020). Alcohol consumption and alcohol-related problems during the COVID-19 pandemic: a narrative review. Australas. Psychiatr..

[6] Delgado-Ortiz L., Carsin A.E., Merino J., Cobo I., Koch S., Goldberg X. (2022). Changes in population health-related behaviors during a COVID-19 surge: a natural experiment. Ann. Behav. Med..

[7] Pollard M.S., Tucker J.S., Green H.D. (2020). Changes in adult alcohol use and consequences during the COVID-19 pandemic in the US. JAMA Netw. Open.

[8] Silczuk A. (2020). Threatening increase in alcohol consumption in physicians quarantined due to coronavirus outbreak in Poland: the ALCOVID survey. J. Public Health.

[9] Hennein R., Lowe S. (2020). A hybrid inductive-abductive analysis of health workers' experiences and wellbeing during the COVID-19 pandemic in the United States. PLoS One.

[10] Greenberg N., Weston D., Hall C., Caulfield T., Williamson V., Fong K. (2021). Mental health of staff working in intensive care during Covid-19. Occup Med (Lond)..

[11] Rond Md (2018). Gedragsregel Nul is de norm formeel vastgelegd KNMG: KNMG.

[12] Good Surgical Practice. Royal College of Surgeons.

[13] Drug, Alcohol and Substance Misuse Policy. https://www.solent.nhs.uk/media/1255/drug-alcohol-and-substance-misuse-policy.pdf:NHS.

[14] Practicing and operating while impaired Royal Australasians College of Surgeons: Royal Australasians College of Surgeons; [Available from: https://www.surgeons.org/about-racs/position-papers/practicing-and-operating-while-impaired-2017#:∼:text=The%20Royal%20Australasian%20College%20of,impaired%20by%20drugs%20or%20alcohol.

[15] Gilligan C., Anderson K.G., Ladd B.O., Yong Y.M., David M. (2019). Inaccuracies in survey reporting of alcohol consumption. BMC Publ. Health.

[16] Boniface S., Kneale J., Shelton N. (2014). Drinking pattern is more strongly associated with under-reporting of alcohol consumption than socio-demographic factors: evidence from a mixed-methods study. BMC Publ. Health.

[17] Garnett C., Crane D., West R., Michie S., Brown J., Winstock A. (2015). Normative misperceptions about alcohol use in the general population of drinkers: a cross-sectional survey. Addict. Behav..

[18] Cunningham J.A., Neighbors C., Wild T.C., Humphreys K. (2012). Normative misperceptions about alcohol use in a general population sample of problem drinkers from a large metropolitan city. Alcohol Alcohol.

[19] Agency EAS. Blood Alcohol Concentration Limits for General Aviation Pilots updated 12-04-2018. Available from: https://www.google.com/url?sa=i&rct=j&q=&esrc=s&source=web&cd=&cad=rja&uact=8&ved=0CAIQw7AJahcKEwjQ1ODxo83_AhUAAAAAHQAAAAAQAg&url=https%3A%2F%2Fad.easa.europa.eu%2Fblob%2FEASA_SIB_2018_07.pdf%2FSIB_2018-07_1&psig=AOvVaw3Pi3ogkSLJuv0VMvI4eHZI&ust=1687193523183823&opi=89978449.

[20] Alcohol & Flying A deadly combination [Available from: https://www.faa.gov/pilots/safety/pilotsafetybrochures/media/alcohol.pdf.

[21] Taylor H.L., Dellinger J.A., Schilling R.F., Richardson B.C. (1983). Proceedings of the Human Factors Society Annual Meeting.

[22] Ross L.E., Mundt J.C. (1988). Multiattribute modeling analysis of the effects of a low blood alcohol level on pilot performance. Hum. Factors.

[23] Legal BAC limits per country (2018). https://apps.who.int/gho/data/view.main.54600.

[24] Blood Alcohol Concent (BAC) (2021). Drink Driving Limits across Europe: European Transport Safety Council.

[25] Research O. (2022).

[26] Mandrioli M., Inaba K., Piccinini A., Biscardi A., Sartelli M., Agresta F. (2016). Advances in laparoscopy for acute care surgery and trauma. World J. Gastroenterol..

[27] Nguyen K.T., Marsh J.W., Tsung A., Steel J.J., Gamblin T.C., Geller D.A. (2011). Comparative benefits of laparoscopic vs open hepatic resection: a critical appraisal. Arch. Surg..

[28] Galaal K., Donkers H., Bryant A., Lopes A.D. (2018). Laparoscopy versus laparotomy for the management of early stage endometrial cancer. Cochrane Database Syst. Rev..

[29] Hajibandeh S., Hajibandeh S., Gumber A.O., Wong C.S. (2016). Laparoscopy versus laparotomy for the management of penetrating abdominal trauma: a systematic review and meta-analysis. Int. J. Surg..

[30] Dorafshar A.H., O'Boyle D.J., McCloy R.F. (2002). Effects of a moderate dose of alcohol on simulated laparoscopic surgical performance. Surgical Endoscopy And Other Interventional Techniques.

[31] Kirby G., Kapoor K., Das-Purkayastha P., Harries M. (2012). The effect of alcohol on surgical skills. Ann. R. Coll. Surg. Engl..

[32] LaparoscopyBoxx [Available from: https://laparoscopyboxx.com.

[33] Bökkerink G.M.J., Joosten M., Leijte E., Verhoeven B.H., de Blaauw I., Botden S. (2021). Take-home laparoscopy simulators in pediatric surgery: is more expensive better?. J. Laparoendosc. Adv. Surg. Tech..

[34] Joosten M., Bökkerink G.M.J., Verhoeven B.H., Botden S. (2023). Evaluating the use of a take-home minimally invasive surgery box training for at-home training sessions before and during the COVID pandemic. J. Laparoendosc. Adv. Surg. Tech..

[35] Joosten M., Hillemans V., Bökkerink G.M.J., de Blaauw I., Verhoeven B.H., Botden S. (2023). The feasibility and benefit of unsupervised at-home training of minimally invasive surgical skills. Surg. Endosc..

[36] Alcolvisor. Mercury Alcovisor (2023). https://www.alcovisor.com/product.php?pid=3&cid=1.

[37] Ali A., Rasheed A., Siddiqui A., Naseer M., Wasim S., Akhtar W. (2015). Non-parametric test for ordered medians: the Jonckheere terpstra test. Int. J. Stat. Med. Res..

[38] Bonferroni C. (1936).

[39] Griswold M.G., Fullman N., Hawley C., Arian N., Zimsen S.R.M., Tymeson H.D. (2018). Alcohol use and burden for 195 countries and territories, 1990–2016: a systematic analysis for the Global Burden of Disease Study 2016. Lancet.

[40] Hanssen I., Smith Jacobsen I.L., Skråmm S.H. (2020). Non-technical skills in operating room nursing: ethical aspects. Nurs. Ethics.

[41] Youngson G.G., Flin R. (2010). Patient safety in surgery: non-technical aspects of safe surgical performance. Patient Saf. Surg..

[42] Bouchery E.E., Harwood H.J., Sacks J.J., Simon C.J., Brewer R.D. (2006). Economic costs of excessive alcohol consumption in the U.S. Am. J. Prev. Med..

[43] Rehm J., Gmel G.E., Gmel G., Hasan O.S.M., Imtiaz S., Popova S. (2017). The relationship between different dimensions of alcohol use and the burden of disease-an update. Addiction.

[44] Murray C.J.L., Aravkin A.Y., Zheng P., Abbafati C., Abbas K.M., Abbasi-Kangevari M. (2020). Global burden of 87 risk factors in 204 countries and territories, 1990–2019: a systematic analysis for the Global Burden of Disease Study 2019. Lancet.

[45] Burton R., Sheron N. (2018). No level of alcohol consumption improves health. Lancet.

[46] (2016). UK Chief Medical Officers' Low Risk Drinking Guidelines.

[47] Perkins A.E., Varlinskaya E.I., Deak T. (2019). From adolescence to late aging: a comprehensive review of social behavior, alcohol, and neuroinflammation across the lifespan. Int. Rev. Neurobiol..

[48] Ramos-Vera C., Serpa Barrientos A., Calizaya-Milla Y.E., Carvajal Guillen C., Saintila J. (2022). Consumption of alcoholic beverages associated with physical health status in adults: secondary analysis of the health information national trends survey data. J Prim Care Community Health.

[49] Amiri S., Behnezhad S. (2020). Alcohol use and risk of suicide: a systematic review and Meta-analysis. J. Addict. Dis..

[50] Keyes K.M., Allel K., Staudinger U.M., Ornstein K.A., Calvo E. (2019). Alcohol consumption predicts incidence of depressive episodes across 10 years among older adults in 19 countries. Int. Rev. Neurobiol..

